# /l/ velarisation as a continuum

**DOI:** 10.1371/journal.pone.0213392

**Published:** 2019-03-11

**Authors:** Susana Rodrigues, Fernando Martins, Susana Silva, Luis M. T. Jesus

**Affiliations:** 1 School of Health Sciences, University of Algarve (ESSUAlg), Faro, Portugal; 2 CLUL, School of Arts and Humanities, University of Lisbon, Lisbon, Portugal; 3 Neurocognition and Language Research Group, Center for Psychology at University of Porto, Faculty of Psychology and Education Science at University of Porto, Porto, Portugal; 4 School of Health Sciences (ESSUA), University of Aveiro, Aveiro, Portugal; 5 Institute of Electronics and Informatics Engineering of Aveiro (IEETA), University of Aveiro, Aveiro, Portugal; Pacific Lutheran University, UNITED STATES

## Abstract

In this paper, we present a production study to explore the controversial question about /l/ velarisation. Measurements of first (*F*_*1*_), second (*F*_*2*_) and third (*F*_*3*_) formant frequencies and the slope of *F*_*2*_ were analysed to clarify the /l/ velarisation behaviour in European Portuguese (EP). The acoustic data were collected from ten EP speakers, producing trisyllabic words with paroxytone stress pattern, with the liquid consonant at the middle of the word in onset, complex onset and coda positions. Results suggested that /l/ is produced on a continuum in EP. The consistently low *F*_*2*_ indicates that /l/ is velarised in all syllable positions, but variation especially in *F*_*1*_ and *F*_*3*_ revealed that /l/ could be “more velarised” or “less velarised” dependent on syllable positions and vowel contexts. These findings suggest that it is important to consider different acoustic measures to better understand /l/ velarisation in EP.

## Introduction

The alveolar lateral approximant has been attracting growing attention in speech research. One reason to this is that phonetic manifestations of the lateral approximant /l/ are characterised by considerable variability across and within languages and dialects, which results in contradictory positions about /l/ velarisation in the literature. In European Portuguese (EP), the lateral is traditionally associated with non-velarised (light) /l/ in syllable onset and velarised (dark) /l/ in coda position (categorical view), but more recent acoustic and articulatory studies considered the existence of velarised realisations of /l/ in both onset and coda positions [1–8]. The present study is concerned with /l/ velarisation in EP and reports acoustic data of /l/ in different vowel contexts and syllable positions. To lay the groundwork for the discussion, several acoustic measures and a variety of linguistic contexts that have not been considered in EP before, are now explored. What is the influence of the syllable position on the acoustic properties of the realisations of /l/? What is the effect of the vowel context on the acoustic properties of the realisations of /l/? Which acoustic properties could be considered to clarify the /l/ velarisation behaviours in EP? The present study explicitly addresses these questions.

### Light and dark realisations of /l/

The distinction between dark /l/ (also known as velarised or pharyngalised /l/) and light /l/ (also known as non-velarised or clear /l/) has long been observed, and the two variants have traditionally been classified as allophones of the same phoneme [9,10].

From an articulatory point of view, lateral sounds typically engage lingual contact along the midsagittal line of the vocal tract, with airflow around one or both sides of the tongue [11–13]. The main articulatory differences between dark /l/ and light /l/ include increased tongue-root retraction and/or increased posterior tongue body for the dark realisation [14,15]. The production of light /l/ involves tongue body raising and fronting, as well as the formation of a single large cavity behind the dentoalveolar constriction. In contrast, dark /l/ is produced with different degrees of tongue predorsum lowering [14,16].

Articulatory differences between light and dark realisations (tongue body configuration and cavity distribution) reflect into different acoustic properties. Although this has been widely acknowledged in the literature, the specific effects of cavity affiliation remain controversial, i.e., little is known yet on how the tongue body configuration and cavity distribution impact the acoustic properties of laterals [17–19]. Still, available principles point to influences on the *frequencies of the first three formants*: We know that the first formant frequency (*F*_*1*_) generally depends more on the back cavity volume than on the volume of other cavities [17] and correlates with tongue height. Thus, considering the volume reduction of the posterior cavity for dark /l/ production, higher *F*_*1*_ values are expected for dark /l/ than for light /l/ [20]. Second, in accordance to the Acoustic Theory of Speech Production, the frequency of the second formant (*F*_*2*_) increases with tongue body fronting [17]. Therefore, the tongue body retraction in dark /l/ production lowers *F*_*2*_ [17,20,18], which means that *F*_*2*_ varies inversely with velarisation degree. Finally, the third formant frequency (*F*_*3*_) is front-cavity dependent and can be conditioned by lip rounding [17,21,22], but it is nevertheless important to characterise the lateral approximant /l/ [22–25].

Variation in *F*_*2*_ frequency has been considered the main acoustic correlate of the difference between light and dark allophones: Light /l/ has a relatively high *F*_*2*_ (and low *F*_*1*_), whereas dark /l/ typically has a low *F*_*2*_ (and high *F*_*1*_) [11,22,26–29]. However, in accordance with an articulatory and acoustic study about American English [30], the strongest correlate of velarisation was tongue body lowering, and acoustic differences were mainly manifested in *F*_*1*_ (not necessarily lowering of *F*_*2*_). The authors emphasised that a higher *F*_*1*_ can emerge as a primary acoustic correlate of velarisation depending on the specific vowel contexts. The third formant frequency is also higher for dark /l/ than light /l/ whenever the velarised realisation is articulated more anteriorly than the non-velarised realisation [17].

Recasens [31] reported formant frequency data for /l/ in 23 languages/dialects, including European Portuguese (EP). The range of mean values was 240–550 Hz for *F*_*1*_, 750–2000 Hz for *F*_*2*_ (*F*_*2*_ exceeds 1500 Hz for light /l/ and has values around 1000 Hz for dark /l/) and 2300–3000 Hz for *F*_*3*_. However, it is known that /l/ may be produced with different degrees of velarisation depending on different factors [1,21,26,31–35]: Language; syllable position; vowel context; individual variation.

Acoustic correlates of velarisation go beyond formant frequencies, since light and dark lateral approximants also show *distinct formant transitions* (formant trajectories generated by articulatory movements) [11,36,37]: Light /l/ show shorter transitions and dark /l/ tends to show slower and smoother transitions (longer transitions may reflect relatively slow dorsal gestures while shorter transitions reflect relatively fast apical gestures) [38]. This illustrates how single acoustic measures may limit the characterisation of /l/ [36]. Nevertheless, a few studies about /l/ production have provided formant transition data (duration, frequency and/or slope). The data presented by Dalston [39], the only available study about liquid consonants that considered the slope of *F*_*2*_ transitions revealed mean values of the slope of *F*_*2*_ transitions of 11.4 Hz/ms for /l/ in syllable onset. However, other syllable positions were not analysed, and, although different vowel contexts were included in the corpus, the data referred only to global mean values.

The different phonetic realisations of alveolar lateral /l/ are characterised not only by the place of articulatory gesture and by the different shapes of the tongue, but also by *coarticulatory resistance*. Coarticulatory resistance describes the lack of influence of adjacent vowel contexts on different phonetic realisations of /l/. Variations in coarticulatory resistance are widely described in the literature and are pointed out as one of the distinctive features between light /l/ and dark /l/. Generally speaking, dark /l/ is more coarticulatory resistant than light /l/. In other words, the coarticulatory effects decrease with the increase of velarisation degree [22,27,33,35,40–43]. Recasens et al. [35,44] compared light /l/ (in German or Italian) and dark /l/ (in Eastern Catalan or American English) and found that the velarised realisation allows less coarticulation. The vowel context has a gradual effect on the velarisation degree [22,33]: Dark /l/ become less dark in the context of a high front vowel (such as /i/), while light /l/ may become less light in the context of a high and back vowel (such as /u/).

The coarticulatory distance associated with dark /l/ (by comparison to light /l/) is particularly evident when the adjacent vowel is /i/ (characterised by tongue body raising and fronting), given the fact that the tongue assumes opposite configurations for the vowel and dark /l/ production. The same is not true when the vowel context is /a/, since the tongue configuration for the vowel and dark /l/ production is quite similar [31,33,40,44–46].

### Categorical versus continuum view of /l/ Velarisation

Languages and dialects differ as to whether they exhibit light /l/ or dark /l/. In the last decades, several studies about articulatory and/or acoustic characteristics present an allophonic perspective about the production of /l/, assuming the existence of a binary distinction between light and dark /l/ (*categorical view*). The dark /l/ appears in coda position and the light /l/ in syllable onset [11,15,23,27,47,48], as noted above.

A number of studies put forward the hypothesis that the degree of velarisation of in the alveolar lateral consonant does not progress categorically, but on a continuum across languages/ dialects [1,22,31–35,40,49]. Recasens and Espinosa [22], analysed data from many studies, and were able to divide languages and dialects into three groups: “(a) Several languages and dialects exhibit a strongly dark variety of /l/ in all positions” (e.g., American English); “(b) Larger acoustic differences as a function of syllable position occur in dialects where /l/ is as dark or less dark than in those listed under (a)” (e.g., British English); “c) Languages and dialects with a clear variety of /l/ have been reported to exhibit the same or a highly similar realization of the alveolar lateral syllable-initially and syllable-final” (e.g., Spanish and German)”.

In a classic study of English /l/, based on acoustic and articulatory data, Sproat and Fujimura [34] argued that there are no distinct allophones of /l/, and that variation is associated with phonetic principles. The phonological /l/ is phonetically implemented as a lighter or darker realisation depending on factors like the position within the syllable and the phonetic duration of the prosodic context containing /l/. They proposed that the phonological /l/ involves two gestures: A vocalic dorsal gesture (attracted to the nucleus of the syllable) and a consonantal apical gesture (attracted to the margin). Sproat and Fujimura [34] found that the /l/ in longer rhymes is darker. Their explanation was that, when the rhyme is short, the tongue dorsum gesture may not have enough time to reach its full target and therefore the /l/ is lighter. However, Huffman [32] showed that a longer /l/ is not always articulated more posteriorly and suggested that the relation between duration and backness can be complicated by differences in coarticulatory effects of neighbouring context or by speaker specific characteristics. On the other hand, the results obtained by Yuan and Liberman [50,51] showed that there is a categorical distinction between light and dark /l/ in American English. These results are consistent with traditional descriptions (/l/ in onset position is light, and /l/ in coda position is dark). Nevertheless, the same authors point out that intervocalic /l/ can be either light or dark, depending on the stress of the vowels, as reported by Huffman [32].

The validity of the gradual hypothesis is also supported by several authors [22,31,33,35,41,49,52] that, having studied various languages, observed different degrees of /l/ velarisation for particular dialects (with light /l/ and dark /l/), regardless of syllable position. In agreement with data reported in Recasens and Espinosa’s [22] study, about two Catalan dialects (Valencian and Majorcan) position-dependent degrees of velarisation are not universal. Their results indicated that /l/ is not necessarily darker in coda position than syllable onset. Thus, consistent with data for languages and dialects exhibiting a very dark realisation of /l/ (e.g., American English), Majorcan /l/ showed no substantial articulatory and acoustic differences, namely in *F*_*2*_ and *F*_*1*_ values.

This articulatory and acoustic data reveal that /l/ velarisation does not proceed categorically but gradually (as a continuum), so languages with one of the two /l/ types (light /l/ or dark /l/) may show lighter or darker consonant qualities [33]. Thus, for example, among languages or dialects with a dark variety of /l/ in all positions, it seems relevant to know the characteristics of /l/ within the continuum.

### European portuguese

As shown above, the articulatory and acoustic properties of /l/ have been well studied for many languages, especially in English [14,15,22,31,34,51], but, in EP, this is a topic that needs more investigation. It is also important to test consistently the different linguistic contexts of /l/ in a controlled way. Although there are recent studies on this topic for EP, it is still not clear how the velarised /l/ behaves within the continuum view.

For the last two decades, it has been argued that /l/ in EP is categorically associated with a non-velarised allophone in syllable onset and a velarised allophone in coda position, based on impressionistic observations [9,53]. However, this point of view is not consensual, especially when considering the empirical descriptions that show the existence of velarised realisations of /l/ in all syllable positions [1–4,6–8,54]. The evidence that /l/ velarisation also occurs in syllable onset was also described in older EP phonetic descriptions [55–57]. In agreement with empirical descriptions of EP lateral /l/, based on acoustic data previously presented by Andrade [1], Recasens and Espinosa [22] concluded that EP belongs to a group of sound systems where /l/ essentially shows the same dark realisation in onset and coda position. It is important to note that Andrade’s [1] study focused only on /l/ realisations in onset and complex onset syllable positions.

Findings from articulatory and acoustic EP studies suggests that /l/ is consistently dark even in the onset position, and that the light versus dark syllabic position distinction is quite subtle (i.e., hard to perceive) and can be influenced by factors such as speaker, vowel context and word position [1,4,54,58]. Generally speaking, acoustic studies have reported *F*_*2*_ values lower than 1500 Hz in all syllable positions (characteristic of /l/ velarisation) but none of them provided specific characteristics about velarisation degrees [1–3,8,54]. However, there are several limitations in these studies (e.g., data collected from a small number of speakers and contexts; different methodological procedures) that preclude more generalised conclusions and leave questions unanswered. For *F*_*2*_, Marques [3] reported that there are different velarisation degrees but does not clearly define the /l/ behaviour in syllable onset and coda positions. In Monteiro’s [8] study, no significant differences were found between syllable positions for *F*_*2*_ values, although slightly higher absolute values for coda position were observed. Oliveira et al. [54] observed higher *F*_*2*_ values for /l/ in intervocalic position, followed by the initial and final position, with statistically significant differences between the intervocalic and final positions. These results suggest a higher velarisation degree in the final position. Nevertheless, these studies considered different syllable and word positions, so it is not clear what is happening in terms of velarisation.

The syllable position effect is also observed in *F*_*1*_ values, although little emphasis is given to this topic in EP studies. However, Marques [3] reported *F*_*1*_ values slightly higher for syllable onset than for coda (medial and final) positions, with statistically significant differences between them. On the other hand, Monteiro [8] observed that *F*_*1*_ values are significantly higher in complex onset than in syllable onset and coda positions. These apparent contradictory *F*_*1*_ behaviour as a function of syllable position, does not clarify its correlation with /l/ velarisation in EP.

The great variability associated with /l/ realisations is also conditioned by the characteristics of the adjacent segments, especially the vowel context, as already explained. The highest *F*_*2*_ values are reached in /i/ vowel context [1,2,8,54], a tendency only contradicted by Marques [3] whose results show that the vowel /i/ has an intermediate effect between /a/ and /u/ contexts. Andrade [2] reports a greater *F*_*2*_ variability in /i/ context, justified by the antagonistic articulatory configurations between dark /l/ and vowel /i/. Regarding *F*_*1*_, it is shown that /l/ assumes distinct values as a function of vowel context in the following progression [3,8]: /a/ > /i/ > /u/.

The acoustic measures most frequently used to differentiate these allophones in EP have been based on *F*_*2*_ [1–3,8,54] and sometimes on *F*_*1*_ values [3,8]. Third formant frequency values and formant transitions information have not been provided for EP.

In the current study, measurements of *F*_*1*_, *F*_*2*_, *F*_*3*_, and the slope of *F*_*2*_ transitions will be analysed to highlight differences in /l/ darkness degree. Analysis of formant frequencies are sensitive to modifications in tongue shape and constriction place, so the degree of /l/ velarisation can be inferred from the acoustic data. Furthermore, the slope of the *F*_*2*_ transition provides dynamic information which also reflects the adjustments in vocal tract shape during speech sound production and various coarticulation effects [47] which may influence the velarisation degree.

### Research questions and hypothesis

The purpose of this paper is to provide further knowledge on the acoustic properties of EP /l/ in order to shed a new light on the controversial question about /l/ velarisation. Data for languages or dialects in which the consonant is typically velarised show differences in velarisation degree depending on several factors, as mentioned before. Therefore, it is expected that this study will contribute to a better understanding of the /l/ velarisation in EP. The main objective is to test the validity of the continuum hypothesis, i.e., if the velarised /l/ shows continuum / non-categorical phonetic properties. We have no reason to doubt the consensus in the literature [22] about the range of mean values for formant frequencies (*F*_*1*_, *F*_*2*_ and *F*_*3*_), especially with regard to *F*_*2*_ (*F*_*2*_ exceeds 1500 Hz for light /l/ and has values around 1000 Hz for dark /l/) and so we make no predictions with respect to these values. However, we predicted differences in the acoustic properties that could support the continuity hypothesis and allow the definition of the velarisation progression from “less velarised” to “more velarised”.

The following research questions, exploring differences in the formant frequencies (*F*_*1*_, *F*_*2*_ and *F*_*3*_) and in the slope of the *F*_*2*_ transitions depending on the syllable position and the vowel context, were posed:

1. What is the influence of the syllable position on the acoustic properties of the realisations of /l/?
2. What is the effect of the vowel context on the acoustic properties of the realisations of /l/?
3. Which acoustic properties could be considered to clarify the /l/ velarisation behaviour in EP?

We therefore tested the following four hypotheses:

1. There are velarised realisations of /l/ in all syllabic positions. /l/ will have low *F*_*2*_ in all syllable positions and /l/ will be more coarticulatory resistant.

2. There is a main effect of the syllable positions in all vowel contexts. In the coda position, /l/ will have higher *F*_*1*_, lower *F*_*2*_ and higher *F*_*3*_ than in onset positions.

3. There is a main effect of the vowel context in all syllable positions. In [i, e, ε] contexts /l/ will have higher slope of the *F*_*2*_ transitions than in other vowel contexts, in [a] context /l/ will have higher *F*_*1*_ than in other vowel contexts, in [i, e, ε] context /l/ will have lower *F*_*1*_ and higher *F*_*2*_ than in other vowel contexts and in [u, o, ɔ] context /l/ will have lower *F*_*2*_ and lower *F*_*3*_ than in other vowel contexts.

However, because EP belongs to a group of sound systems where /l/ essentially shows the same dark realisation in onset and coda position [22] and dark /l/ is more resistant to coarticulatory effect than light /l/ [31,33,43,59], we predicted that formant frequencies of /l/ in the three different vowel contexts would exhibit less variation, especially *F*_*2*_ values.

4. There is an interaction between vowel contexts and syllable positions. It is expected that /l/ will have higher *F*_*1*_ in coda position and in [a] context and that /l/ will have lower *F*_*2*_ in coda position and in [u, o, ɔ] context.

## Materials and methods

An independent ethics committee (Comissão de Ética da UICISA-E, Coimbra, Portugal) gave specific approval for this study. All speakers agreed to participate in the study and gave their written informed consent.

### Speakers

Five adult female and five adult male speakers, whose ages ranged from 20 to 40 years, were recruited. They were all monolingual speakers of a southern variant of EP, and none had history of speech and/or language disorders.

The participants filled in a detailed questionnaire, designed to capture information about their personal characteristics. Speakers were assessed by a Speech and Language Therapist to make sure they had typical speech (based on informal conversation) as well as typical orofacial structures (face, tongue and dental arch morphology and the face and tongue functions). They were also assessed by an Audiologist and none of them had hearing loss.

None of the speakers was aware of the purpose of the study and none of them had phonetic training. All speakers agreed to participate in the study and gave their written informed consent.

### Corpus design

The set of 20 real words selected for data collection had the following characteristics: Trissyllabic words–although this is not the most frequent word extension in EP [60], it allows the lateral consonant to occupy the middle of the word in onset, complex onset and coda positions; paroxytone stress pattern–the most frequent position for word accent in EP [60]. In addition, the stressed syllable coincided with the syllable with /l/.

There is a relationship between the stress pattern and the spectral difference between adjacent consonants and vowels, with a stronger energy difference between the consonant and the vowel if the vowel is stressed [61], which facilitates the acoustic analysis.

The vowel context varied between the seven oral vowels /i, e, ε, u, o, ɔ, a/. All EP oral vowels may occur in the stressed position, except for the vowel [ɨ] that results from weakening processes of tonic vowels. Also, there are no words with the vowel [ɐ] in tonic position, since, as for vowel [ɨ], this vowel occurs mostly in an unstressed position [62]. For this reason, there are no stimuli associated with vowels [ɐ] and [ɨ].

In complex onset and coda positions, /l/ was preceded and followed, respectively, by a stop consonant, (e.g., <complica> [kõˈplikɐ] or <cobalto> [kuˈbaltu]).

For syllable onset, the choice was the stop /p/, not only because it is more frequent in EP, but also because it is a segment produced with lip rounding that will reduce the interaction of articulatory gestures between the two consonant elements that make up the syllable [1].

In coda, the consonant /t/ was selected to occupy the next position. Although it has the same place of articulation as /l/, it allows combinations with all previously defined vowels, except for the words <pocilgo> and <rebelde>, because no other stimulus satisfied this condition.

It should be noted that there are no real words with the vowel /e/ and lateral /l/ in a medial coda, not even with any other stop consonant.

The initial and final syllables of the selected stimuli were, as far as possible, CV syllables. Due to the difficulty in finding words that met all criteria, we introduced stimuli whose initial syllable did not correspond to the CV sequence, e.g., <explodo>, <inculto> or <envolto>.

Given the impossibility of constructing a minimal pair corpus, in order to obtain a set of stimuli as balanced as possible, considering the various pre-defined criteria, whenever possible, items with only one liquid consonant were considered. Words <templário>, <rebelde>, <revolta>, <liberto> and <lagarto> did not meet this criterion due to the difficulty of finding stimuli that satisfied the set of defined parameters.

Based on the linguistic criteria described above, the words that make up the corpus used in data collection is shown in Table 1. It should be noted that neither the frequency effect of the words nor the type of word (noun, verbs and adjective) were controlled, otherwise the corpus under analysis would have resulted in a very restricted set of words.

**Table 1:** The corpus used in the data collection.

Vowel context	Onset position	Complex onset position	Coda position
/i/	Baliza [bɐˈlizɐ]	Complica [kõˈplikɐ]	Pocilgo [puˈsilɡu]
/u/	Caluda [kɐˈludɐ]	Depluma [dɨˈplumɐ]	Inculto [ĩˈkultu]
/e/	Maleza [mɐˈlezɐ]	Dupleto [duˈpletu]	-
/o/	Balofo [bɐˈlofu]	Explodo [ʃˈplodu]	Envolto [ẽˈvoltu]
/ε/	Maleta [mɐˈlεtɐ]	Completo [kõˈplεtu]	Rebelde [ʀɨˈbεldɨ]
/a/	Palato [pɐˈlatu]	Templário [tẽˈplaɾju]	Cobalto [kuˈbaltu]
/ɔ/	Galocha [ɡɐˈlɔʃɐ]	Simplote [siĩˈplɔtɨ]	Revolta [ʀɨˈvɔltɐ]

### Data collection

Speakers produced 100 utterances (5 random repetitions of 20 different words). Each word was produced within the frame sentence <Diga a palavra… por favor> [ˈdiɡapɐˈlavɾɐ… puɾfɐˈvoɾ] (“say the word… please”). The use of frame sentences favours the more natural pronunciation in comparison with words spoken in isolation [63,64]. The same frame sentence was used repeatedly for all corpus words, so that the influence of the acoustic and linguistic context on the corpus words was controlled.

Stimuli were randomly organised into five separate sets and later presented in *MS Power Point 2010*, one sentence on each slide to avoid a “list effect” reading. Each sentence was displayed for six seconds on the monitor of a computer used to present the frame sentences located outside the soundproof booth, where the recordings took place.

Before the recordings started, speakers were instructed to read the sentences at a natural rhythm. A short pause was made after every five repetitions so that speakers could rest. The total recording time did not exceed 35 minutes.

Data were recorded at the University of Algarve in Faro, Portugal, in a soundproof booth, using a DPA 4006-TL microphone located 30cm in front of the speaker’s lips, connected to an audio interface (TASCAM US-800) and a desktop computer located outside the soundproof booth. The acoustic signal was recorded at 16 bits and a sampling frequency of 44100 Hz using *Audacity* 2.0.

### Corpus annotation

The uncompressed .WAV files, corresponding to each of the repetitions, were organised into individual files (each of which corresponds to the word production inserted in the frame sentence).

All target words were segmented and annotated manually using both spectrographic and sound wave display in Praat 5.3.30 [65]. Although this process is somewhat subjective, it is essential that the segmentation is consistent. Thus, the segmentation of the acoustic signal was performed according to criteria proposed by several authors [10–12,47,49,64,66,19], as presented below.

**Fig 1:**
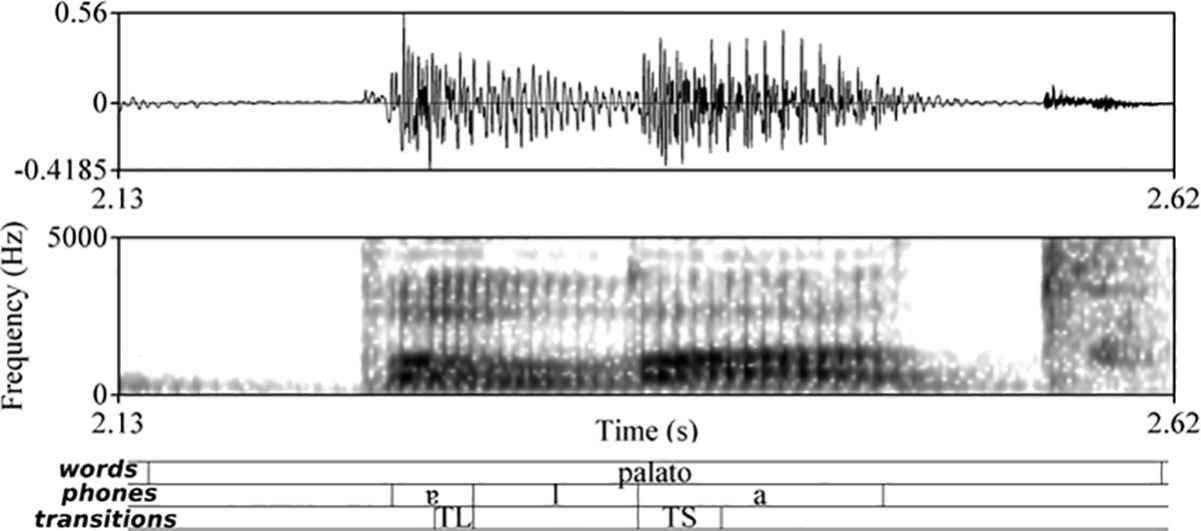
Annotation of the acoustic signal in the word <palato> [pɐˈlatu] produced by speaker FS.

The waveforms and spectrograms (Fig 1) of all the corpus words were analysed to detect the start of preceding vowel (onset and coda) or stop consonant (complex onset)–phone 1 (*phones* tier in Fig 1). The start of the vowel was marked when well-defined frequencies of the first three formants were present, by the onset of periodicity in the waveform and corresponding onset of glottal pulses in the spectrogram [11,12,64,67] and checked against the spectrogram for the visible presence of *F*_*2*_ [66]. The start of the stop consonant was considered to occur when there was a beginning of white space after the end of glottal pulses of the preceding vowel [12,18,64,67,68].

The start of transition into /l/ (*transitions* tier (TL) in Fig 1) was considered to occur when there was a decrease in amplitude and the end of steady state in the vowel, i.e., the part of the sound where the formants are relatively steady in frequency and unaffected by transitions from neighbouring sounds [10,49]; the end of a visible *F*_*2*_ in the spectrogram also indicated the end of the vowel [66].

The start of transition into /l/ in complex onset was marked at the end of the voice onset time [11,12,64,67,68] and was determined by the beginning of the characteristics of a lateral approximant /l/, i.e., a drop in intensity relative to that of the neighbouring segments, regularity of the waveform and clearly visible formants [12,18,25,64,19].

The end of transition into /l/ (the start of steady state in the lateral / phone 2 –*phones* and *transitions* tiers in Fig 1) coincided with the start of the steady state in the lateral /l/ [49].

The start of transition out of /l/ (*phones* and *transitions* tiers–the start of phone3 and TS interval in Fig 1) was marked by an increase in amplitude and the end of steady state in the lateral.

The end of transition out of /l/ (the start of steady state in vowel in the *transitions* tier) and the end of the following vowel (onset or complex onset) or stop consonant (coda), i.e., phone 3 in the *phones* tier in Fig 1, were also annotated.

The main cues to identify the lateral /l/ were low amplitude, identifiable formant structures, spectral continuity, and a period of steady state formant patterns, combined with formant shifting to and from adjacent speech sounds [12,25,64,19,67,69].

Three annotation levels were therefore defined: Words, phones and transitions. In Fig 1, the first tier is the phonological tier, where the target word is annotated using Portuguese orthography. The second one is the phonetic tier where the realisation of the target, based on visual inspection of the waveform and spectrogram, is annotated using the International Phonetic Alphabet and segmentation of the target word based on the criteria mentioned earlier in this section. The third tier involves segmentation of the transitions. Transitions were considered as belonging to the vocalic segments, following the examples and arguments provided by Clark, Yallop and Fletcher [10].

### Acoustic data analysis

Formant data was extracted using the following Praat 5.3.40 function: To Formant (burg) … 0.01 5 5500 0.025 50; [Time step(s), Max. number of formants, Maximum formant (Hz), Window length(s), Pre-emphasis from (Hz)]–split Levinson algorithm.

Matlab R2007b was used for post-processing the data initially extracted using Praat 5.3.40, with the following formant frequency threshold values: 200 Hz < *F*_*1*_ < 900 Hz; 700 Hz < *F*_*2*_ < 2600 Hz; 1600 Hz < *F*_*3*_ < 3900 Hz; 2800 Hz < *F*_*4*_ < 5000 Hz. These limits were defined based on the literature [1,3,23–25,32,39,54,70] in order to eliminate eventual outliers resulting from errors introduced by the automatic extraction. All productions that were not within limits were excluded from the analysis.

The following parameters at specific instances and time intervals (Fig 1) were considered for analysis: *F*_*1*_, *F*_*2*_ and *F*_*3*_ at the middle of phone 1 (t1FF), phone 2 (t2FF) and phone 3 (t3FF); *F*_*2*_ at the start of the transition into the lateral (t4FF); *F*_*2*_ at the end of the transition into the lateral (t5FF); *F*_*2*_ at the start of the transition out of the lateral (t6FF); *F*_*2*_ at the end of the transition out of the lateral (t7FF); absolute values (Hz/ms) of slopes *F*_*2*_ from the nuclear vowel into (coda) and out (onset and complex onset) of the laterals, i.e., (t5FF-t4FF)/dur2 and (t7FF-t6FF)/dur4, respectively. The slopes could be either positive or negative, depending on the direction of *F*_*2*_ movement. For statistical analysis, only absolute values were used. A similar procedure was adopted by Kent [71].

The database for statistical analysis included 645 /l/ realisations (only canonical /l/ realisations were selected for statistical analysis): 220 in onset; 217 in complex onset; 208 in coda. We used R software to run linear mixed-models analysis on each dependent variable (frequency of *F*_*1*_, *F*_*2*_, *F*_*3*_, and slope of *F*_*2*_ transition). Analyses were carried out with the lme4 package [72] for R [73].The *lmerTest* package [72] was used for significance values, and *sjPlot* for summary tables [72]. Vowel context (front, back and front/central) and syllable position (onset, complex onset and coda) were used as fixed factors, and speaker, item and repetition as crossed random factors (random intercepts). The grouping of vowels in front [i, e, ε], front/central [a] and back [u, o, ɔ] vowels followed criteria proposed in previous studies [3,22,74,75].

In order to test for the main effects of each factor (vowel context and syllable position) as well as the interaction between the two, we created a series of models. These models allowed the comparison between the full model (containing all terms of interest, e.g., vowel and syllable effects plus interaction) and the reduced model (lacking the component of interest, e.g., vowel and syllable effects, for testing the interaction). The main effects of syllable position and vowel context were tested against the null model (intercept-only), which worked as a reduced model in these cases. We ran the *Anova* function for model comparison (significance test provided by Chi-square) and relied on the Akaike Information Criterion (AIC) to determine whether the term of interest would either increase or decrease explanatory value (lower AIC values indicate increased explanatory power). Taking the results in Table 2 as an illustration, the Chi-square values for the comparison of models with a vowel effect term (full model) vs. without this term (reduced model, null in this case) show significant differences (*p* = .006). As we inspect AIC values, we saw that the full model had a lower value than the reduced one. This indicated that the full model had increased explanatory power compared to the reduced one, meaning that there was a significant vowel effect.

**Table 2:** Descriptive statistics for *F*_*1*_ (in Hz) at the midpoint of the steady state.

Descriptives									
	Onset			Complex Onset			Coda		
Vowel context	N	Mean	SD	N	Mean	SD	n	Mean	SD
F: [i, e, ε]	97	377.79	52.96	93	420.54	86.29	86	435.85	98.60
B: [u, o, ɔ]	77	387.35	62.62	84	398.18	54.24	79	459.72	87.60
FC: [a]	46	447.78	67.81	40	448.10	72.82	43	554.77	77.24
Total	220	395.77	65.36	217	416.96	74.71	208	469.50	100.55

F, front; B, back; FC, front/central

In case of significant main effects with no further interaction, we performed pairwise comparisons across the three levels of the independent variable holding the effect (syllable and/or vowel), using Bonferroni corrections for multiple comparisons (significance value multiplied by three). In case of significant interactions, we ignored main effects and broke down the analysis into syllable effects per vowel type, and into the reverse approach–vowel effects per syllable position. Again, pairwise comparisons were made using Bonferroni corrections. Unless otherwise specified, the significance level was .05.

## Results

### First formant frequency values

Table 2 provides a general overview of *F*_*1*_ mean values at the midpoint of the steady state of /l/; the *F*_*1*_ mean values vary in the progression coda > complex onset > onset; the first formant frequency at the midpoint of the lateral alveolar is higher in [a] context than in other vowel contexts.

There was a main effect of vowel context (Table 3), with higher *F*_*1*_ values for [a] compared to both front and back vowels. The effect of syllable position did not reach significance, nor did the interaction between vowel context and syllable position.

**Table 3:** Effects on *F*_*1*_ (in Hz) at the midpoint of the steady state. Local effect size measures such as partial eta-squared do not apply to linear mixed effects models [76]. R^2^ measures may be used instead as whole-model effect sizes for the purpose of comparison and meta-analysis [77].

Effects				
Effect	AICs reduced/full model		Comparison test reduced vs. full model (Chi-square; *p*)	R^2^ Full model
Syllable	7137.9	7137.4	4.48; .11 (ns)	.595
Vowel	7137.9	7131.6	10.27; .006**	.593
Syllable x Vowel	7129.9	7135.9	2.05; .72 (ns)	.599
**Pairwise comparisons** (direction: *p/*corrected *p*, R^2^)				
Vowel FC > F: .006/.018* & FC > B: .004/.012*, .598				
Number of observations = 645				

F, front; B, back; FC, front/central

** < .01

* < .05; ns–non-significant

### Second formant frequency values

Table 4 summarises the frequencies of *F*_*2*_ at the midpoint of the steady state of /l/. The results show very similar mean values for all syllable positions (Table 3).

**Table 4:** Descriptive statistics for *F*_*2*_ (in Hz) at the midpoint of the steady state.

Descriptives									
	Onset			Complex Onset			Coda		
Vowel context	n	Mean	SD	N	Mean	SD	n	Mean	SD
F: [i, e, ε]	97	1018.20	125.06	93	1108.67	249.96	86	1040.01	141.07
B: [u, o, ɔ]	77	1025.21	335.95	84	953.31	123.39	79	1048.18	298.46
FC: [a]	46	947.70	94.35	40	1006.05	138.39	43	973.19	96.90
Total	220	1005.91	220.82	217	1029.61	202.91	208	1029.30	210.91

F, front; B, back; FC, front/central

The main effects of vowel context and syllable position were not significant, nor was the interaction between the two (Table 5).

**Table 5:** Effects on *F*_*2*_ (in Hz) at the midpoint of the steady state.

Effects				
Effect	AICs reduced/full model		Comparison test reduced vs. full model (Chi-square; *p*)	R^2^ full
Syllable	8662.1	8666.1	0.03; .98 (ns)	.237
Vowel	8662.1	8666.6	2.52; .28 (ns)	.234
Syllable x Vowel	8667.5	8668.4	7.11; .13 (ns)	.231
Number of observations = 645				

F, front; B, back; FC, front/central; ns–non-significant

### Third formant frequency values

Table 6 summarises the *F*_*3*_ values at the midpoint of the lateral’s steady state. The third formant frequency is higher for coda position, followed by onset and complex onset positions. The highest values are associated with the back vowel context [u, o, ɔ].

**Table 6:** Descriptive statistics for *F*_*3*_ (in Hz) at the midpoint of the steady state.

Descriptives									
	Onset			Complex Onset			Coda		
Vowel context	n	Mean	SD	N	Mean	SD	N	Mean	SD
F: [i, e, ε]	97	2831.37	263.91	93	2854.55	255.45	86	2969.40	272.88
B: [u, o, ɔ]	77	3064.30	287.38	84	2986.50	338.04	79	3147.89	253.45
FC: [a]	46	2915.67	313.58	40	2870.42	307.45	43	3048.21	281.36
Total	220	2930.52	300.15	217	2908.55	304.40	208	3053.48	277.83

F, front; B, back; FC, front/central

There were significant main effects of vowel context, syllable position, and a significant interaction between the two (Table 7). When breaking down the interaction and considering syllable effects for each vowel context, we saw increased values in coda compared to complex onset for the three vowel contexts. Additionally, there were increased values in coda compared to onset for the vowels [i, e, ε, a] but not for the vowels [u, o, ɔ]. Regarding the complementary analysis–vowel effects per syllable position (vowel per syllable, Table 7), back vowels showed significant or marginally-significant higher *F*_*3*_ values than [i, e, ε, a] at onset and complex onset positions. At coda positions, back vowels surpassed the [i, e, ε] context, but not the [a] context.

**Table 7:** Effects on *F*_*3*_ (in Hz) at the midpoint of the steady state.

Effects				
Effect	AICs reduced/full model		Comparison test reduced vs. full model (Chi-square; *p*)	R^2^ Full
Syllable	8784.2	8779.0	9.21; .01*	.553
Vowel	8784.2	8773.3	14.93; < .001**	.554
Syllable x Vowel	8755.7	8668.4	95.30; < .001**	.231
**Pairwise comparisons** (direction: *p/*corrected *p*, R^2^)				
Syllable per vowel F: C > O, .006/.018* & C > CO, .030/.090º, .451 B: C > CO, .003/.009**, .677 FC: C > O, .003/.009* & C > CO, < .001***, .589 Vowel per syllable O: B > F, < .001*** & B > FC, < .001***,.497 CO: B > F, .033/.099º & B > FC, .005/.015*, .683 C: B > F, .011/.033*				
Number of observations = 645				

F, front; B, back; FC, front/central; O, onset; CO, complex onset; C, Coda

*** < .001

** < .01

* < .05

º < .10; ns–non-significant

### Slope of the *F*_*2*_ transition

**Fig 2:**
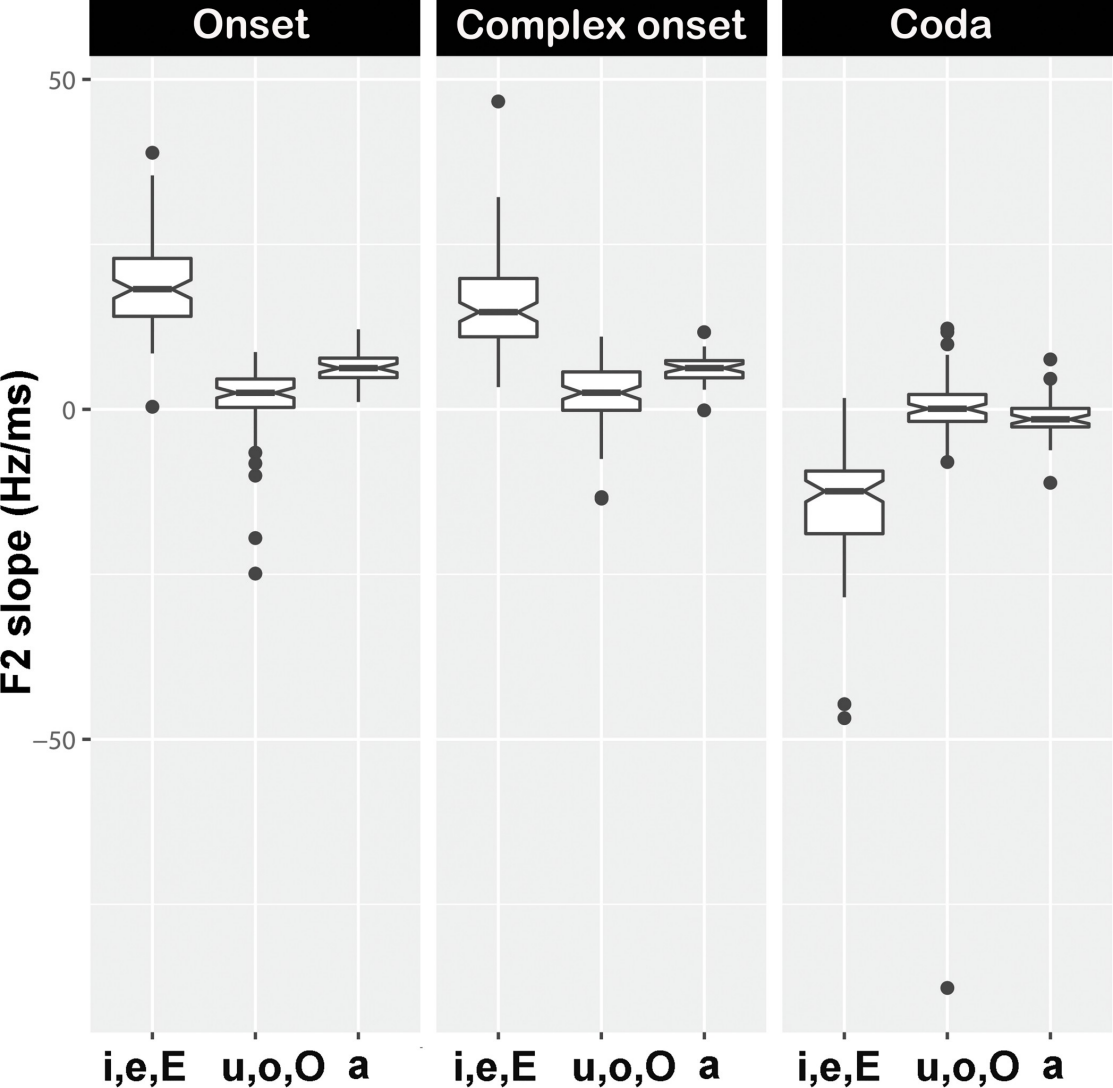
Slope of *F*_*2*_ transition for [i, e, ε], [u, o, ɔ] and [a] contexts in all syllable positions.

Table 8 summarises the mean values for slope of the *F*_*2*_ transition. The slope of the *F*_*2*_ transition was negative (downward direction) in coda position for all vowel contexts (Fig 2). In onset and complex onset positions, the slope of the *F*_*2*_ transition was positive (upward direction). For all syllable positions, the slope of the *F*_*2*_ transitions is more pronounced in [i, e, ε] contexts (Table 8). The lme analysis showed a significant main effect of vowel context (Table 9), with front vowels eliciting higher values than [a, u, o, ɔ], in all syllable positions.

**Table 8:** Descriptive statistics for mean slope of *F*_*2*_ transition (Hz/ms) in onset, complex onset and coda positions.

Descriptives									
	Onset			Complex Onset			Coda		
Vowel context	n	Mean	SD	N	Mean	SD	N	Mean	SD
F: [i, e, ε]	97	18.87	6.38	93	15.86	7.06	86	-14.61	7.98
B: [u, o, ɔ]	77	1.41	5.48	84	2.23	4.56	79	-.52	10.77
FC: [a]	46	6.31	2.35	40	6.15	2.30	43	-1.34	3.11
Total	220	10.17	9.64	217	8.80	8.35	208	-6.52	10.88

F, front; B, back; FC, front/central

**Table 9:** Effects on mean slope of *F*_*2*_ transition (Hz/ms) at the midpoint of the steady state.

Effects				
Effect	AICs reduced/full model		Comparison test reduced vs. full model (Chi-square; *p*)	R^2^ Full
Syllable	4130.9	4134.1	0.85, .65 (ns)	.607
Vowel	4130.9	4100.2	34.71, < .001***	.607
Syllable x Vowel	4102.1	4107.5	2.64, .62 (ns)	.607
**Pairwise comparisons** (direction: *p/*corrected *p*, R^2^)				
Vowel F > B, < .001*** & F > FC, < .001***, .607				
Number of observations = 645				

F, front; B, back; FC, front/central

*** < .001; ns–non-significant)

The small number of participants could have influenced some of the null effects. In the future, a more powerful database could result in positive findings.

## Discussion

The present study of /l/ production in different syllable positions (onset, complex onset and coda) and in different vowel contexts ([i, e, ε, a, u, o, ɔ]) tested the validity of the continuum hypothesis in which the velarised /l/ shows gradual, non-categorical phonetic properties. We predicted a main effect of syllable position in all vowel contexts and a main effect of vowel context in all syllable positions. It was also predicted that syllable position would interact with vowel context, as observed in at least for some acoustic measures.

### The effect of syllable position

#### First formant (*F*_*1*_)

The mean *F*_*1*_ values obtained in the present study are in agreement with results from previous experimental studies, performed mostly for American English, which show higher *F*_*1*_ values in coda position [24,27,35,70,78]. However, these differences were small, which could justify the absence of statistically significant differences between syllable positions in our study showing that velarisation degree across syllable positions could not be inferred by *F*_*1*_ values. Previous EP studies [3,8] revealed a main effect of syllable position on *F*_*1*_ values, but their apparent contradictory behaviour as a function of syllable position does not show the impact of velarisation on *F*_*1*_ values. Additionally, these studies also reported an interaction between syllable position and vowel context, but did not provide more details about it. Nevertheless, in contrast with previous acoustic studies about /l/ velarisation [22,32], the present study shows that there is no interaction between syllable position and vowel context for *F*_*1*_ values.

#### Second formant (*F*_*2*_)

The results of this study are consistent with the low *F*_*2*_ values (around 1000 Hz) obtained by previous studies and point to a strongly dark realisation of EP /l/ in all word-positions and vowel contexts [1,3,8,31]. However, the explanation for /l/ velarisation as a continuum solely based on *F*_*2*_ values breaks down here, since /l/ produced in various syllable positions and vowel contexts were not statistically different and there was no interaction between vowel context and syllable position. For EP, the absence of significant differences between onset and coda position had already been reported for *F*_*2*_ [3,8], although slightly higher absolute values for coda position had been observed. Nevertheless, Oliveira et al. [54] reported significant higher *F*_*2*_ values for /l/ in intervocalic position than for /l/ in final position. Due to methodological differences, especially in relation to corpus design and statistical analysis procedures, it is difficult to make proper comparisons.

Considering the results obtained for American English, which is part of the group of languages (including EP) with dark /l/ in all syllable positions [22], it has been observed that the mean of *F*_*2*_ values are lower in coda (post-vocalic and final-word) than in syllable onset (pre-vocalic, intervocalic and word-initial), although in none of the syllable positions *F*_*2*_ values are higher than 1000 Hz [70,78]. The mean *F*_*2*_ values obtained in the present study are in line with these observations. However, the differences observed between syllable positions are small and do not coincide with the trend towards lower values in coda, which was also observed in other EP studies [3,8]. Despite these observations, only Oxley’s et al. [70] study showed significant differences between the word-initial (onset) and word-final (coda) positions, but not between the syllable onset and coda in an intervocalic context. These results are, to a certain extent, in accordance with our observations for /l/ in syllable onset and coda position in the middle of the word.

The results obtained in the present study are also in line with Recasens and Espinosa’s [22] analyses of Majorcan (a Catalan dialect with velarised realisations of / l / in all syllable positions), showing the absence of statistically significant differences between /l/ in word-initial and /l/ in word-final for the *F*_*2*_ values. A similar tendency was pointed out by Recasens and Farnetani [35] for Italian and English. It should be noted that the comparison between the results of the present study and the ones obtained by Oxley et al. [70], Recasens and Espinosa [22] and Recasens and Farnetani [35], should be regarded with caution, since there are differences in the characteristics of the stimuli used, namely those concerning word position.

#### Third formant (*F*_*3*_)

Some authors [22,24,25,78] consider that *F*_*3*_ values are important to characterise liquid consonants, but there are few studies that explored the link between this acoustic measure and /l/ velarisation [22,24,78]. For EP, as far as we know, no previous study considered this variable.

The data collected and analysed in the present study shows that the lateral /l/ in coda position presented higher *F*_*3*_ values than in other syllable positions. Regarding the mean values obtained, these results are in accordance with data reported in previous studies [24,39]. However, other authors [23,25,79] present lower *F*_*3*_ frequency values (between 1600 and 2600 Hz), and the differences between pre-vocalic (onset), intervocalic (onset at the middle of the word) and post-vocalic (coda) relations reported by Espy-Wilson [23] are small (pre-vocalic: 2533Hz; intervocalic: 2640 Hz; post-vocalic: 2630 Hz). In spite of the absence of statistical processing of data, Lehman and Swartz [24] showed that the /l/ in coda position is characterised by higher *F*_*3*_ values when compared to /l/ in syllable onset, which is compatible with our findings.

Regarding previous studies comparing Catalan [22] and English [80] dialects that exhibit a dark or a light variety of /l/, it has been observed that dialects with velarised /l/ in all syllable positions generally exhibit higher *F*_*3*_ values than dialects with non-velarised /l/ realisations. Identical results are reported by Recasens [31]. If, on the one hand, our data corroborate the existence of velarised /l/ in all syllable positions in EP (since the mean *F*_*3*_ values are within the same order of magnitude as previous studies), on the other hand the main effect of the syllable position in vowel context indicates that /l/ in coda position has a higher velarisation degree than in onset and complex onset positions, except in [u, o, ɔ] context where no statistically significant differences have been identified between coda and syllable onset. This, however, cannot be discussed in the context of other studies that analysed /l/ in different positions [22,23] because they do not refer the existence of statistically significant differences between syllable positions for *F*_*3*_ values.

Generally speaking, since these results are not clearly supported by the literature, it would be essential to compare acoustic and articulatory data, since the *F*_*3*_ values seem to be inversely related with the size of the anterior cavity and further conditioned by lip rounding [17,21,22]. The establishment of relations between acoustic and articulatory information will allow to understand which cavity is effectively related with *F*_*3*_ values and, thus, to provide more enlightening explanations as to the obtained results.

#### Slope of the *F*_*2*_ transition

Although we observed neither a main effect of syllable position nor interaction between syllable position and vowel context on the slope of *F*_*2*_ transition, another relevant finding has arisen. According to the results of the present study, when /l/ occurs in coda position the slope of *F*_*2*_ transition is negative, regardless of the vowel context considered. This behaviour indicates that, in this syllable position, the /l/ is produced with greater tongue retraction than the adjacent vowels. Although no important effect of the syllable position on the slope of *F*_*2*_ transition has been found, these observations may help us better understand the velarisation progression from “less velarised” to “more velarised”, based on the assumption that a velarised /l/ is produced with tongue body retraction.

### The effect of vowel context

#### First formant (*F*_*1*_)

Regarding the influence of the vowel context on the *F*_*1*_ values, the results reported in the present study show that /l/ assumes values statistically different as a function of the vowel context, but there is no interaction between vowel context and syllable position. The more detailed analysis of the mean values obtained confirms that *F*_*1*_ is higher in the context of [a] (low vowel which is also characterised by higher *F*_*1*_ values) and lower in the contexts of [i, e, ε] and [u, o, ɔ] vowels. The higher *F*_*1*_ values associated with [a] vowel contexts have been consistently reported by several authors for /1/ in different syllable positions [22,24,31,32,35]. This is an unsurprising behaviour, since high *F*_*1*_ values are also characteristic of this vowel, which may explain the observed effect of the vowel context on the lateral. For EP, statistically significant differences between *F*_*1*_ values for /a/ > /i/ > /u/ context have also been reported in the literature [3,8], partly supporting the trend observed in the present study. The absence of significant differences between the contexts [i, e, ε] and [u, o, ɔ] observed in the present study may reflect constraints imposed by the methodological option adopted to group the vowel contexts with reference to tongue advancement (anterior, central and posterior) and not to the height of the tongue dorsum. Although only the main effect of the vowel context on the *F*_*1*_ values has been observed, this finding is relevant to show that in [a] vowel context, /l/ is produced with tongue lowering. Thus, considering the relationship between *F*_*1*_ values and /l/ velarisation degree, our acoustic data suggested the existence of a higher velarisation degree in [a] context than in [i, e, ε] and [u, o, ɔ] contexts.

#### Second formant (*F*_*2*_)

With respect to the main effect of the vowel context in all syllable positions, our expectations have not been completely confirmed, namely for *F*_*2*_ values. Our initial expectation was motivated by previous studies [33,35] that show that even linguistic systems with velarised realisations of /l/ in all syllable positions report differences in *F*_*2*_ values motivated by the adjacent vowel context, and they share the fact that the higher values are associated to the vowel /i/ context. A similar result has been recently been reported by Simonet [43].

However, the absence of significant differences between the vowel contexts under study, suggests that the adjacent vowel context does not influence the *F*_*2*_ values, showing coarticulatory resistance associated with /l/ in all syllable positions. Therefore, the findings of the present study are in line with previous results that support the arguments presented by Recasens et al. [22,40,42,44] according to which /l/ velarisation is correlated with degree of coarticulatory resistance for this consonant. This finding is also consistent with the assumption that dialects with strongly dark /l/ should not exhibit two positional allophones [22], reinforced the hypothesis of /l/ velarisation as a continuum and not as a categorical property.

#### Third formant (*F*_*3*_)

The main effect of the vowel context in all syllable positions for *F*_*3*_ values seems to be mainly associated with the [u, o, ɔ] context; lip rounding in EP is only observed in posterior vowel production [69]. The *F*_*3*_ values are significantly higher in this vowel context, for all syllable positions, contradicting the tendency that the lip rounding mainly lowers *F*_*3*_ values [21]. Although Lehman and Swartz [24] and Recasens and Espinosa’s [22] studies are not directly comparable (because they focus on different languages and use different methodological procedures), their results also seem to indicate the opposite direction. Data reported by Lehman and Swartz [24] for pre-vocalic /l/ and post-vocalic /l/ showed very consistent *F*_*3*_ values for /l/ in different vowel context, suggesting the absence of an important effect of the vowel context for this dependent variable. In Recasens and Espinosa [22], despite the lack of statistical analysis of variable *F*_*3*_, it was reported that non-velarised /1/ seems to be more sensitive to rounding of the lips, since the *F*_*3*_ values for Valencian (non-velarised Catalan dialect) are lower in /u/ vowel context (between 1400 Hz and 1800 Hz) and higher for /i/ and /a/ contexts (between 2075 Hz and 2755 Hz). The same trend is not reported for Majorcan (velarised Catalan dialect), whose *F*_*3*_ values range from 2500 Hz to 3300 Hz, and /l/ seems to be less sensitive to the effect of vowel context. Although both studies have collected acoustic and articulatory data, specifically Electropalatographic (EPG) data, and consistent relationships have been observed between the two reports, none of the studies provides robust relationships with the rounding of the lips (the aspect closely related to the *F*_*3*_ values). It is true that the EPG data do not provide this information. However, in the particular case of the study by Lehman and Swartz [24] whose objective was to clarify the effect of the vowel context on the /l/ production based on acoustic and articulatory data, this explanation would be important.

The results reported in the present study contradict, to some extent, the general trend that rounding lowers all formant frequencies, especially *F*_*3*_ [21] and our initial prediction, because statistical analysis showed that *F*_*3*_ values are significantly higher in back vowel contexts, for all syllable positions. The effect of vowel context on *F*_*3*_ values raises the following questions: Is it possible that there is an articulatory “similarity” between back vowels and velarised /l/ that eliminates the lip rounding effect? Is lip rounding responsible for the higher /l/ velarisation degree? To clarify these issues in the future, it is important to combine acoustic and articulatory data.

Considering the differences found between the present study and previous studies, it is important to note that there are considerable differences not only between languages, but also between characteristics of stimuli used for data collection and methodological procedures in general, so comparisons and generalisations should be cautious.

In summary, and according to information obtained from the *F*_*3*_ values, EP /l/ seems to be sensitive to lip rounding, even if it is in the opposite direction when compared whit previous studies [21,22,24].

#### The slope of the *F*_*2*_ transition

Results obtained for the slope of the *F*_*2*_ transitions indicate only a main effect of vowel context for all syllable positions. As expected, in [i, e, ε] contexts /l/ showed higher slope of the *F*_*2*_ transitions than for other vowel contexts, for all syllable positions. The occurrence of a steeper slope of *F*_*2*_ transition in anterior vowel context denotes a greater articulatory contrast between these vowels and /l/ regardless of syllable position. Our results thus seem to reflect the antagonistic nature of the lingual configuration that characterises the [i, e, ε] vowels and velarised /l/ (e.g., the tongue dorsum is raised and fronted for /i/ and lowered and backed for dark /l/), as reported in several studies [31,33,40,44–46). This explanation obviously needs to be supported by more acoustic data for more speakers (including also other dialects of EP) and, possibly, by performing a vowel analysis based on articulatory data. The articulatory study of Martins et al. [81], revealed some differences in tongue body configuration between laterals and vowels, but these differences are not specified, invalidating any kind of more detailed relation that can be established with the data of the present study.

An important point to note is the high standard deviation values for the slope of the *F*_*2*_ transition; on several situations, they are higher than the mean values obtained. This is likely a results of variation associated to speaker individual characteristics, a justification which is widely supported by previous acoustic and/or articulatory studies [26,31–34]. This could also be related with difficulties experienced during data segmentation, especially in the exact identification of the boundaries between /l/ and back vowels. In such specific conditions, it is natural that the measures taken to calculate the slope of *F*_*2*_ transition may be subject to greater variability. Similar difficulties have been previously reported by Lee-Kim, Davison and Hwang [30].

## Conclusions

The study presented here provides novel contributions to the acoustic description of EP /l/, using new acoustic measures (such as *F*_*3*_ and the slope of the *F*_*2*_ transition) to describe /l/ velarisation. In most cases, velarisation is not correlated with a single formant but is specified by the joint contribution of the different acoustic measures. It is clear from the results obtained, that it is important to consider acoustic measures such as *F*_*1*_, *F*_*2*_, *F*_*3*_ and the slope of the *F2* transition to better understand /l/ velarisation.

Although the experimental evidence reported in the present paper, namely *F*_*2*_ values, is in support of the idea that /l/ velarisation occurs in all syllable positions (onset, complex onset and coda positions) [41,48,53], the information obtained from *F*_*3*_ values appears to be a strong acoustic correlate that may be considered to clarify the /l/ velarisation behaviour in EP. The higher *F*_*3*_ values in coda position and in [u, o, ɔ] context suggest that /l/ is “more velarised” in these situations. However, the interaction between syllable position and vowel context provides more specific information and indicates that /l/ is “more velarised” when occurs in coda position and in back vowel contexts. The *F*_*1*_ results and the absence of statistical differences for *F*_*3*_ values between [u, o, ɔ] and [a] for coda position indicate that /l/ may be “more velarised” also in [a] vowel context and in coda position the velarisation degree is similar for these vowel contexts.

The slope of the *F*_*2*_ transition is not directly related to /l/ velarisation, but it proved to be relevant to better understand the relationship between /l/ and adjacent vowels. The greater articulatory contrast between [i, e, ε] vowel contexts and /l/ in all syllable positions, together with information obtained from *F*_*1*_ and *F*_*3*_ values, reinforced the idea that /l/ in front vowel contexts in “less velarised”.

Our results clearly indicate that the information obtained from *F*_*3*_ values is important to better understand /l/ velarisation in EP and should be considered in future acoustic studies about /l/ in this language.

To conclude, a continuum /l/ realisations has been observed in EP which is dependent on syllable positions and vowel context, as reported by Sproat and Fujimura [34], but with a smaller scope of variation that what is found in American English. The consistently low *F*_*2*_ suggests that /l/ is velarised in all syllable positions, as noted above, but variation in *F*_*1*_ and *F*_*3*_ suggests that /l/ could be “more velarised” or “less velarised” as a result of several factors.

We are currently considering the following as future work: Including more speakers from different EP dialects, since they can introduce factors that induce variation, and synchronously acquiring acoustic and articulatory data for the EP lateral /l/. This would clarify and assist with the interpretation of the acoustic results.

## Supporting information

S1 Database Excel 2016 database used for statistical analysis and some sound files for exemplification (one file for each speaker with /l/ in different syllabic positions and vowel contexts; only “canonical” realisations of /l/ were included). (ZIP)
